# Supplementary material for: Modeling glioblastoma heterogeneity as a dynamic network of cell states
Source: Mol Syst Biol. 2021 Sep 16;17(9):e10105. doi: 10.15252/msb.202010105 (PMC8444284; doi:10.15252/msb.202010105)
Supplement: Supplementary file 5 — Source Data for Figure 3 [file MSB-17-e10105-s001.zip › Figure3A_sourcedata/GSEA_3065/nosigdb_state2.GseaPreranked.1622718419215/GANDAL_IBD_UP_PMID29439242.html]

Details for gene set GANDAL\_IBD\_UP\_PMID29439242[GSEA]

|  || Dataset | state2 |
| Phenotype | NoPhenotypeAvailable |
| Upregulated in class | na\_neg |
| GeneSet | GANDAL\_IBD\_UP\_PMID29439242 |
| Enrichment Score (ES) | -0.6829849 |
| Normalized Enrichment Score (NES) | -2.3752327 |
| Nominal p-value | 0.0 |
| FDR q-value | 0.0 |
| FWER p-Value | 0.0 |
Table: GSEA Results Summary

  

Fig 1: Enrichment plot: GANDAL\_IBD\_UP\_PMID29439242      
 Profile of the Running ES Score & Positions of GeneSet Members on the Rank Ordered List

  

| PROBE | GENE SYMBOL | GENE\_TITLE | RANK IN GENE LIST | RANK METRIC SCORE | RUNNING ES | CORE ENRICHMENT || 1 | EGR1 |  |  | 1691 | 0.077 | -0.1663 | No |
| 2 | RAB31 |  |  | 1977 | 0.065 | -0.1850 | No |
| 3 | HIF1A |  |  | 2182 | 0.058 | -0.1965 | No |
| 4 | ARFGAP3 |  |  | 2237 | 0.056 | -0.1923 | No |
| 5 | STAT1 |  |  | 2336 | 0.053 | -0.1933 | No |
| 6 | ST3GAL4 |  |  | 2391 | 0.051 | -0.1900 | No |
| 7 | UBE2L6 |  |  | 2901 | 0.036 | -0.2377 | No |
| 8 | IFI16 |  |  | 3054 | 0.033 | -0.2481 | No |
| 9 | SOD2 |  |  | 3366 | 0.026 | -0.2765 | No |
| 10 | ISG20 |  |  | 3567 | 0.023 | -0.2938 | No |
| 11 | RGS2 |  |  | 4038 | 0.015 | -0.3412 | No |
| 12 | WARS |  |  | 4517 | 0.007 | -0.3909 | No |
| 13 | ANGPTL2 |  |  | 5312 | -0.005 | -0.4747 | No |
| 14 | PDE4B |  |  | 5498 | -0.008 | -0.4928 | No |
| 15 | S100A11 |  |  | 5569 | -0.009 | -0.4986 | No |
| 16 | GPX8 |  |  | 6123 | -0.019 | -0.5542 | No |
| 17 | IFITM2 |  |  | 6128 | -0.019 | -0.5513 | No |
| 18 | PDPN |  |  | 6446 | -0.024 | -0.5807 | No |
| 19 | KLHL5 |  |  | 6599 | -0.027 | -0.5920 | No |
| 20 | PHLDA1 |  |  | 6867 | -0.033 | -0.6146 | No |
| 21 | PLAUR |  |  | 6906 | -0.034 | -0.6125 | No |
| 22 | XBP1 |  |  | 6931 | -0.035 | -0.6088 | No |
| 23 | FOS |  |  | 7222 | -0.043 | -0.6321 | No |
| 24 | ANXA1 |  |  | 7265 | -0.044 | -0.6288 | No |
| 25 | PCDH17 |  |  | 7304 | -0.045 | -0.6249 | No |
| 26 | KDELR3 |  |  | 7339 | -0.046 | -0.6203 | No |
| 27 | CDK12 |  |  | 7759 | -0.058 | -0.6545 | No |
| 28 | LPCAT1 |  |  | 8027 | -0.068 | -0.6708 | Yes |
| 29 | SLC7A5 |  |  | 8067 | -0.070 | -0.6625 | Yes |
| 30 | BACE2 |  |  | 8071 | -0.070 | -0.6504 | Yes |
| 31 | RARRES3 |  |  | 8092 | -0.071 | -0.6399 | Yes |
| 32 | SAMD9L |  |  | 8129 | -0.073 | -0.6307 | Yes |
| 33 | PLAU |  |  | 8131 | -0.073 | -0.6177 | Yes |
| 34 | THY1 |  |  | 8139 | -0.074 | -0.6053 | Yes |
| 35 | TRIM22 |  |  | 8154 | -0.075 | -0.5935 | Yes |
| 36 | LY96 |  |  | 8332 | -0.085 | -0.5972 | Yes |
| 37 | FKBP11 |  |  | 8400 | -0.089 | -0.5885 | Yes |
| 38 | PRRX1 |  |  | 8427 | -0.091 | -0.5751 | Yes |
| 39 | C1R |  |  | 8644 | -0.107 | -0.5791 | Yes |
| 40 | PRDM1 |  |  | 8693 | -0.110 | -0.5645 | Yes |
| 41 | IFITM3 |  |  | 8720 | -0.113 | -0.5471 | Yes |
| 42 | GJA1 |  |  | 8728 | -0.114 | -0.5275 | Yes |
| 43 | CD74 |  |  | 8754 | -0.117 | -0.5094 | Yes |
| 44 | TGFBI |  |  | 8761 | -0.117 | -0.4891 | Yes |
| 45 | BHLHE40 |  |  | 8804 | -0.123 | -0.4717 | Yes |
| 46 | IL13RA2 |  |  | 8854 | -0.129 | -0.4538 | Yes |
| 47 | CYR61 |  |  | 8901 | -0.136 | -0.4345 | Yes |
| 48 | CDH11 |  |  | 8983 | -0.146 | -0.4171 | Yes |
| 49 | COL4A1 |  |  | 9284 | -0.226 | -0.4088 | Yes |
| 50 | IGFBP7 |  |  | 9295 | -0.229 | -0.3690 | Yes |
| 51 | CXCR4 |  |  | 9328 | -0.251 | -0.3277 | Yes |
| 52 | TIMP1 |  |  | 9387 | -0.334 | -0.2742 | Yes |
| 53 | TNC |  |  | 9403 | -0.383 | -0.2074 | Yes |
| 54 | GLIPR1 |  |  | 9404 | -0.387 | -0.1384 | Yes |
| 55 | IGFBP5 |  |  | 9438 | -0.797 | 0.0002 | Yes |
Table: GSEA details [plain text format]

  

Fig 2: GANDAL\_IBD\_UP\_PMID29439242: Random ES distribution      
 Gene set null distribution of ES for **GANDAL\_IBD\_UP\_PMID29439242**
